# Lipidomic profiling in metastatic prostate cancer captures tumor metabolic rewiring and its modulation by androgen receptor–targeting therapy

**DOI:** 10.1038/s41391-026-01100-z

**Published:** 2026-03-13

**Authors:** Sara Bleve, Francesco Ravera, Silvia Rodrigues, Filippo Pederzoli, Hubert Pakula, Nicole Brighi, Emilio Francesco Giunta, Giuseppe Schepisi, Alessandra Virga, Giorgia Gurioli, Salvatore Luca Burgio, Giuseppe Nicolo’ Fanelli, Lisa M. Butler, David M. Nanus, Johannes V. Swinnen, Pier Vitale Nuzzo, Cristian Lolli, Ugo De Giorgi, Massimo Loda

**Affiliations:** 1 Department of Medical Oncology, IRCCS Istituto Romagnolo per lo Studio dei Tumori (IRST) “Dino Amadori”, Meldola, Italy; 2 Department of Pathology and Laboratory Medicine, Weill Cornell Medical College, New York, NY USA; 3 Department of Internal Medicine and Medical Specialties, University of Genoa, Genoa, Italy; 4 Biosciences Laboratory, IRCCS Istituto Romagnolo per lo Studio dei Tumori (IRST) “Dino Amadori”, Meldola, Italy; 5 Division of Pathology, Department of Translational Research and New Technologies in Medicine and Surgery, University of Pisa, Pisa, Italy; 6 First Division of Pathology, Pisa University Hospital, Pisa, Italy; 7 South Australian Health and Medical Research Institute, North Terrace, Adelaide, SA Australia; 8 South Australian Immunogenomics Cancer Institute & Freemasons Centre for Male Health and Well-Being, Adelaide University, North Terrace, Adelaide, SA Australia; 9 Department of Medicine, Weill Cornell Medicine, New York, NY USA; 10 Caryl and Israel Englander Institute for Precision Medicine, Weill Cornell Medicine, New York, NY USA; 11 Laboratory of Lipid Metabolism and Cancer, Department of Oncology, Leuven Cancer Institute (LKI) and Leuven Institute for Single Cell Omics (LISCO), KU Leuven, Leuven, Belgium; 12 Department of Experimental Medicine, University of Salento, Fazzi Hospital, Lecce, Italy; 13 Nuffield Department of Surgical Sciences, Lincoln College, University of Oxford, Oxford, UK; 14 Dana-Farber Cancer Institute, Harvard Medical School, Boston, MA USA

**Keywords:** Prognostic markers, Cancer metabolism

## Abstract

**Background:**

Reprogrammed lipid metabolism with massive upregulation of tumor cell-autonomous synthesis of saturated fatty acids is a hallmark of prostate cancer (PCa) and is driven in part by aberrations in androgen receptor (AR) signaling. While lipid alterations are well described in primary PCa, the extent to which the circulating lipidome reflects tumor-associated metabolic changes in metastatic disease, and its role in therapy response, remains to be determined. This study aims to assess whether plasma lipid profiling captures tumor metabolic rewiring, and whether this reflects response to AR-targeting therapy, in metastatic castration-resistant PCa (mCRPC).

**Methods:**

Quantitative plasma lipidomics was performed on plasma samples collected from patients with mCRPC (*n* = 50) and cancer-free subjects (C-FS, *n* = 14). Samples from patients with mCRPC were collected longitudinally at the time of progression on androgen deprivation therapy prior to initiation of first-line enzalutamide (Enza), after the start of treatment with Enza, before progression on Enza.

**Results:**

Compared to C-FS, patients with mCRPC showed distinct lipidomic signatures, characterized by increased levels of monounsaturated lipids and altered composition of the phospholipid and sphingolipid pool, mimicking the aberrations known to occur in primary PCa tissue. Enza treatment markedly reduced total lipid levels, decreased major phospholipid classes and ceramides, while increasing sphingomyelins. Notably, quantitative differences in specific sphingolipid species occurring after Enza treatment correlated with survival outcomes.

**Conclusions:**

Plasma lipidomics reflects key metabolic features of PCa and is profoundly impacted by AR inhibition, with prognostic relevance in patients with mCRPC. These findings support its potential as a non-invasive tool for monitoring disease activity and treatment response, and lay the groundwork for lipid-based biomarkers in mCRPC, while indicating that the lipidomic alterations observed may help inform ongoing and forthcoming research on metabolic targeting.

## Introduction

Prostate cancer (PCa) remains a significant global health burden, particularly in its advanced, treatment-resistant stages [1]. Metastatic castration-resistant prostate cancer (mCRPC) has a 5-year survival rate below 50%, despite advances in therapeutic strategies [2–4]. Among next-generation hormonal agents for mCRPC, enzalutamide (Enza) has demonstrated survival benefits in both chemotherapy-naïve and pre-treated patients [5, 6]. It inhibits key steps in androgen receptor (AR) signaling by blocking androgen binding, preventing AR nuclear translocation, and inhibiting DNA binding.

Metabolic reprogramming in PCa promotes a distinct lipogenic tumor phenotype, marked by increased de novo lipid synthesis, and altered lipid profiles relative to normal prostate tissue [7–11]. Consistently, primary PCa tissue exhibits a lipidomic profile enriched in monounsaturated lipids and elongated fatty acids within specific phospholipid classes, reflecting enhanced lipids synthesis, elongation, and desaturation [12–16]. However, the effects of systemic therapies and in particular of AR-targeting agents on lipid metabolic pathways remain poorly characterized. The majority of lipidomics studies in PCa have relied on tumor tissue analyses, an approach with limited applicability in the metastatic setting where biopsies are rarely feasible. At the same time, studies investigating the circulating lipidome in advanced disease have predominantly focused on correlations between individual lipids species and clinical outcomes, without addressing the broader impact of systemic therapy on lipid metabolism.

In the present study, we performed a comprehensive plasma lipidomic profiling in patients with mCRPC and evaluated longitudinal changes during treatment with Enza. By integrating lipidomic data with clinical parameters and outcome measures, we sought to elucidate the impact of hormonal therapy on systemic lipid metabolic reprogramming.

## Materials and methods

### Patients and study design

The mCRPC cohort included patients from the IRCCS ‘Dino Amadori’ Cancer Institute (Meldola, Italy) initiating Enza as first-line treatment for castration-resistant disease. The diagnosis of mCRPC was established according to Prostate Cancer Clinical Trials Working Group 3 (PCWG3) and RECIST 1.1 criteria. All patients underwent staging with PSMA PET imaging to confirm metastatic disease and assess tumor burden. Blood samples were collected at baseline and at predefined time points during treatment. PSA levels were monitored every 4–6 weeks until death or study cutoff (April 30, 2024). The first patient was enrolled on January 1, 2018.

Cancer-free subjects (C-FS) served as a control group and consisted of men undergoing prostate biopsy for digital rectal examination suspicious for malignancy or elevated prostate specific antigen (PSA) levels at the Presbyterian Hospital (New York, USA), with histopathology confirming absence of malignancy.

### Samples collection and processing

C-FS fasting blood samples were collected immediately prior to prostate biopsy. In patients with mCRPC, fasting blood samples were collected at baseline (after progression on androgen deprivation therapy (ADT) and before the start of Enza) and every 4 weeks during treatment with Enza until radiographic progression. All samples were collected in EDTA tubes, centrifuged at 3000 × g for 10 min. The plasma supernatant was further clarified at 15,000 × g for 10 min. Plasma aliquots were then stored at −80 °C.

### Lipidomics analysis

Lipidomic profiling was performed by Lipometrix (KU Leuven, Belgium) using their liquid chromatography–electrospray ionization tandem mass spectrometry on a Nexera X2 UHPLC system (Shimadzu) coupled with a hybrid triple quadrupole/linear ion trap mass spectrometer (6500 + QTRAP system; AB SCIEX), as previously described [12, 17].

### Statistical analysis

All statistical analyses were conducted in the R Environment (Rstudio version 2024.12.0 + 467.pro1). Lipid concentration values were log-transformed and scaled before all differential analyses. Linear models were built to assess the differences in lipid levels between C-FS and patients with mCRPC. Due to C-FS selection based on sample availability, analyses were adjusted for age—which differed between C-FS and mCRPC—and BMI, used as a proxy for patients’ metabolic status. Paired t-test was used to evaluate lipid changes over time in mCRPC, while Spearman correlation was used to evaluate associations with continuous clinical variables. Elongation indexes were calculated for the main PL classes according to Butler et al. [12]. Briefly, the abundance of each PL species within the same PL class was expressed relatively to the shortest PL species of the same saturation subclass. Linear regression models adjusted for age and BMI were then used to compare elongation indexes between mCRPC and control subjects.

Survival analyses in patients with mCRPC were conducted using Cox regression on scaled data. Univariate and multivariable models were used adjusting for clinically relevant factors, including age, PSA response, Gleason score of the primary tumor, and disease volume at ADT progression according to the CHAARTED trial definition [18]. PSA response was calculated as the ratio of baseline PSA assessed before Enza start to PSA assessed at the closest time point from sample collection after therapy start. Radiographic progression was defined using PCWG3 criteria for skeletal disease and RECIST 1.1 criteria for soft-tissue lesions, and progression-free survival (PFS) was measured from treatment initiation to the date of radiographic progression. Both PFS and overall survival (OS) were measured from ADT progression.

## Results

### Patient characteristics

Baseline plasma samples were available for a total of 64 individuals, including 14 C-FS and 50 patients with mCRPC. Among the mCRPC cohort, 42 patients had additional plasma samples collected after Enza start. For a subset of 7 patients, samples were also obtained at the time of radiological disease progression.

Patients with mCRPC were older than C-FS (*p*-value < 0.0001), while there were no significant differences in BMI or PSA levels (Table 1).

**Table 1:** Baseline characteristics of the study cohorts.

	C-FS (*N* = 14)	mCRPC (*N* = 50)	*p*-value
*Age*, *Median (IǪR)*	60 (53.25 - 63.75)	77 (69 - 81)	<0.0001
*BMI*, *Median (IǪR)*	25 (24.1 - 27.04)	26.49 (24.61 – 28.07)	0.13
*PSA*, *Median (IǪR)*	4.53 (3.2 - 5.57)	2.1 (1.07 - 5.79)	0.23
*Gleason, n (%)* ≤*7* >*7* *Not available*	/	18 (36%) 25 (50%) 7 (14%)	
*FU (y)*	/	3.02 (2.18 - 4.14)	

Data are reported as median and interquartile range (IQR) for continuous variables and as number (percentage) for categorical variables. *PSA*: prostate-specific antigen. *FU* follow-up duration in years. *NA* not available.

In the mCRPC cohort, after a median follow-up of 36.2 months (range = 4.3 - 124.5 months), 24 patients experienced radiographic progression, and 19 patients died. The median radiographic PFS was 28.2 months (range = 2.5 – 76.1 months).

A detailed summary of subject demographics and clinical characteristics is provided in Supplementary Table 1.

### Plasma lipidomic profiling in patients with mCRPC mimics aberrations occurring in primary PCa tissue

**Fig. 1:**
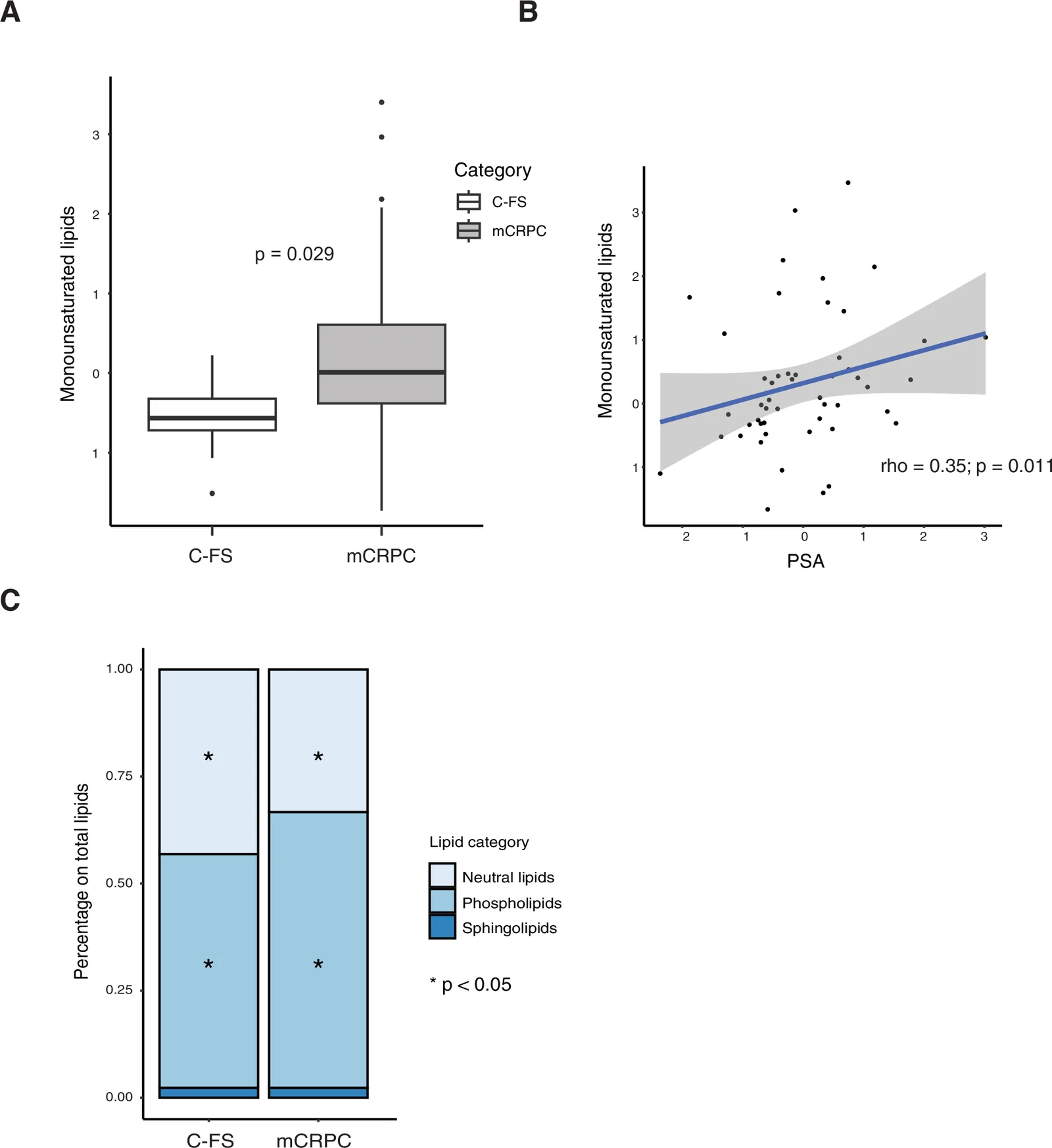
Differences in the overall plasma lipidome in C-FS versus patients with mCRPC. **A** Monounsaturated lipid levels in C-FS versus mCRPC patients. P-values are reported after adjusting for age and BMI. **B** Correlation between PSA levels and monounsaturated lipids in mCRPC patients. **C** Stacked bar plot showing the distribution of lipid classes (neutral lipids, phospholipids, sphingolipids) expressed as a percentage of total lipids in C-FS versus mCRPC patients.

A total of 2017 plasma lipid species belonging to 17 lipid classes were assessed and grouped under the three major categories: PL, sphingolipids (SL), and neutral lipids (triacylglycerols, diacylglycerols, and cholesteryl esters). Raw lipidomics data are available in Supplementary Table 2. Concentration of total monounsaturated lipids was significantly higher in patients with mCRPC, even after adjusting for age and BMI (adjusted *p*-value = 0.029, Fig. 1A). Moreover, the overall amount of monounsaturated lipids positively correlated with PSA levels (Spearman rho = 0.35, *p* = 0.011; Fig. 1B), with a trend towards significant association with PSMA-PET SUV values (Spearman rho = 0.27, *p*-value = 0.059; Supplementary Fig. 1) in patients with mCRPC. Patients with mCRPC were relatively enriched for PL, while being depleted of neutral lipids (Fig. 1C).

**Fig. 2:**
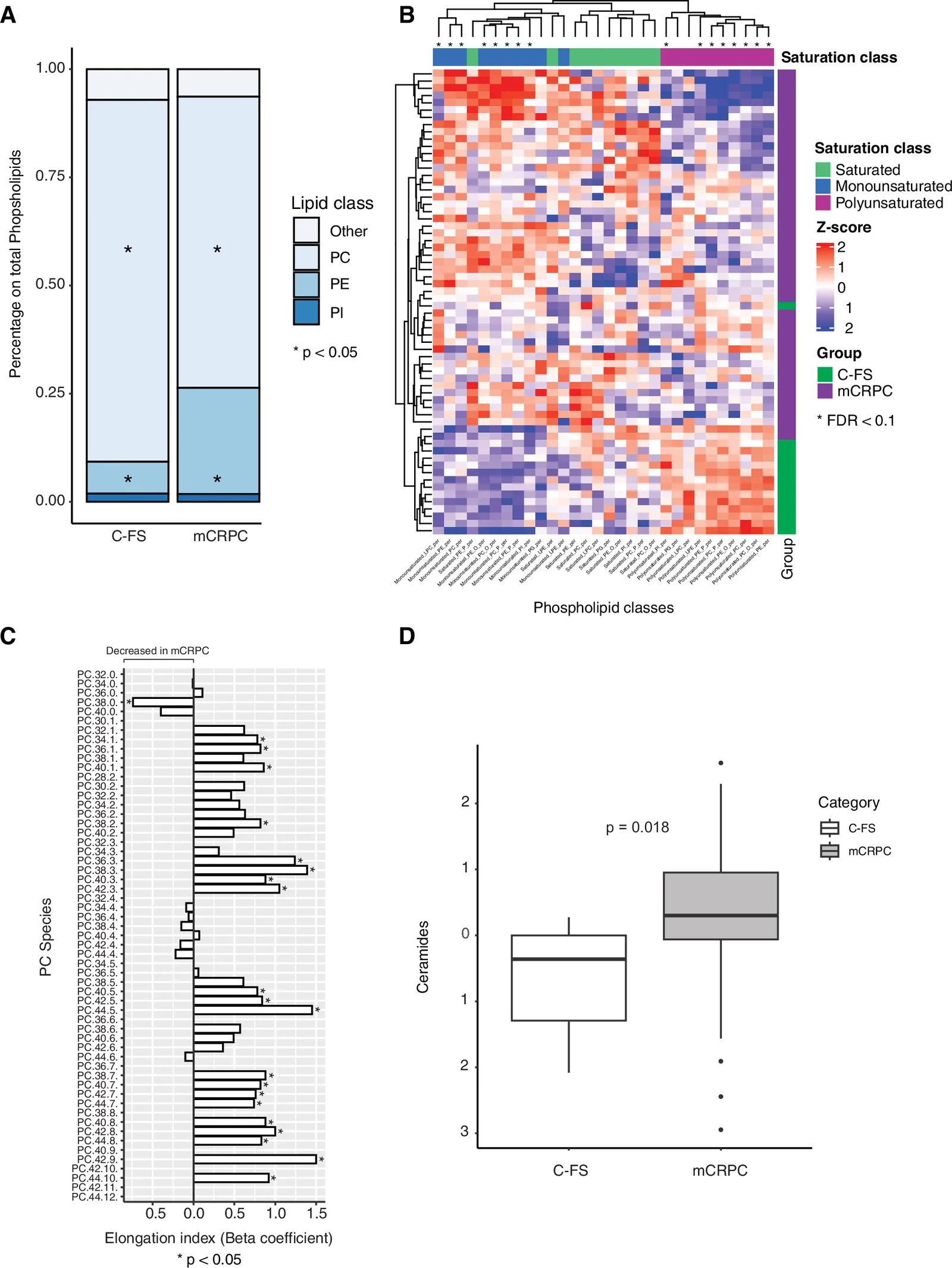
Differences in the plasma phospholipid and sphingolipid pool in C-FS versus patients with mCRPC. **A** Stacked bar plot showing the percentage distribution of the main PL classes, including PC, PE, PI, and minor PL, indicated as “other”. Asterisks indicate statistically significant differences (*p* < 0.05). **B** Heatmap of PL classes grouped by saturation group (i.e. saturated, monounsaturated, polyunsaturated). Columns indicate the different lipid classes, while rows indicate the individual samples. **C** Differences in elongation of PC species in C-FS versus mCRPC patients. Linear models were built to assess the differences in the elongation indexes of PC species in C-FS versus mCRPC patients. Each row represents the beta coefficient of such models and indicates a decreased (left) or an increased (right) elongation of PC species in patients with mCRPC compared to C- FS. **D** Ceramide levels in C-FS and patients with mCRPC.

In both cohorts, the most represented classes within the PL pool were phosphatidylethanolamine (PE), phosphatidylcholine (PC), and phosphatidylinositol (PI). In the mCRPC cohort, a shift in the distribution of major PL species was observed, specifically characterized by a relative increase in PE and a decrease in PC (Fig. 2A). Notably, patients with mCRPC were relatively enriched in monounsaturated PL and depleted for polyunsaturated PL (Fig. 2B). In addition, several saturated PL species also appeared to be increased in mCRPC, although less markedly than monounsaturated species. We also observed substantial elongation in the fatty acyl chain of PL across plasma samples from patients with mCRPC. To quantitatively assess these changes, we calculated an elongation index for each of the main PL species. Elongation indexes were defined as the ratio of the abundance of each PL species to that of the shortest species within its respective saturation subclass [12]. Indeed, an increased fatty acid chain length was observed for most PC and PI species in patients with mCRPC compared to C-FS (Fig. 2C, Supplementary Fig. 2). For PE species, an increased chain length was observed mainly in monounsaturated species (Supplementary Fig. 2).

Significantly higher concentration of ceramides was observed in mCRPC, even after adjusting for age and BMI (adjusted *p* = 0.018, Fig. 2D). No significant differences were observed for the other main SL classes. Similarly, no significant differences were detected across the different lipid classes within neutral lipids between the two study cohorts.

### Treatment with Enza is associated with broad changes in the overall PL and SL pool

To investigate the effects of AR inhibition on systemic lipid metabolism, longitudinal lipidomic profiling was performed in patients with mCRPC before and after treatment with Enza and at radiographic progression.

**Fig. 3:**
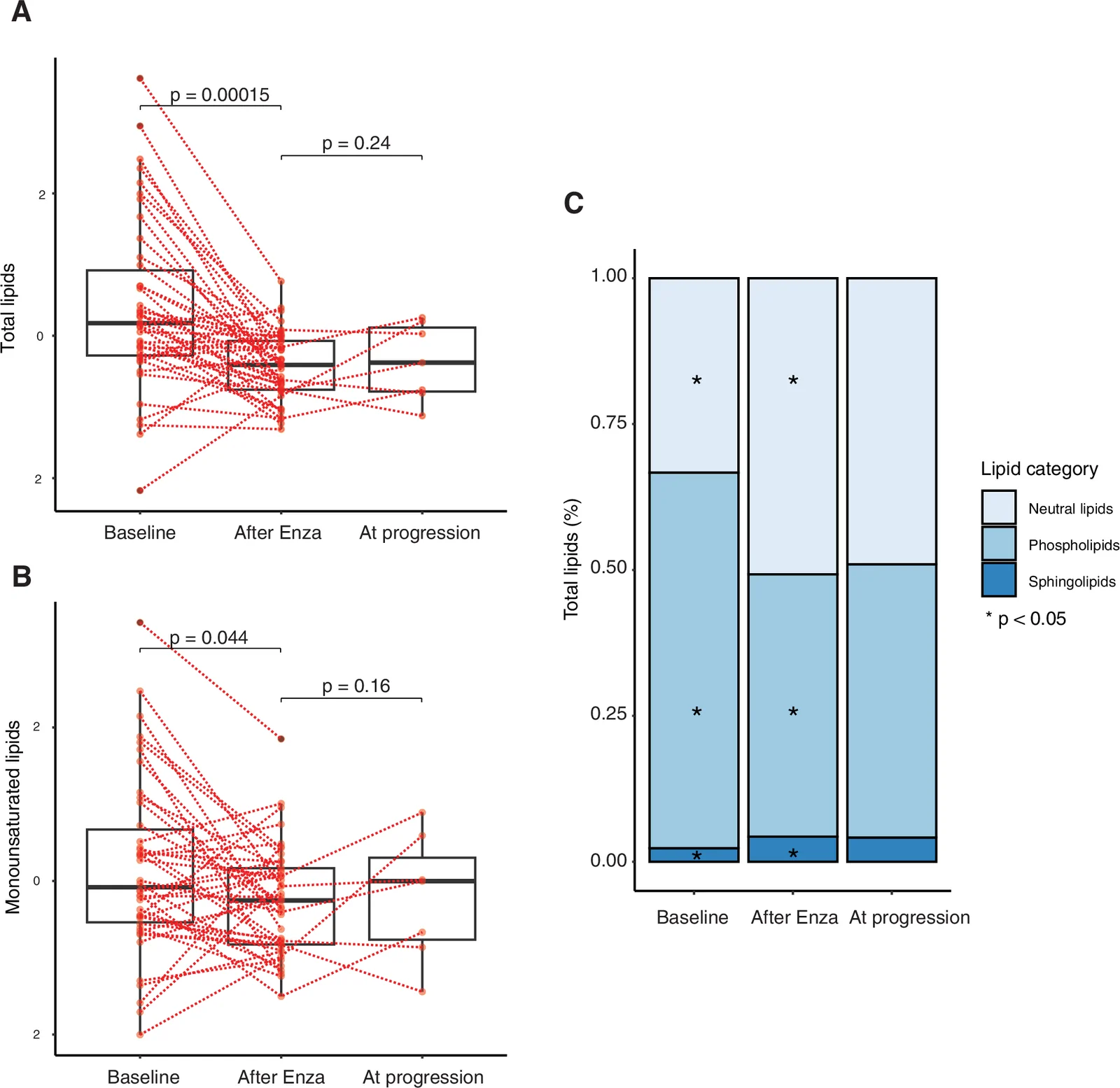
Longitudinal analysis of circulating lipid during Enza treatment and at progression. **A** Paired comparison of total lipid levels and **B** monounsaturated lipids at baseline, after Enza start, and at disease progression. **C** Stacked bar plot showing the relative distribution of lipid categories (neutral lipids, phospholipids, and sphingolipids) as a percentage of total lipids at baseline, after Enza start, and at disease progression. Asterisks indicate statistically significant differences from the previous time point (*p* < 0.05).

Enza treatment resulted in a substantial reduction in total lipid content (*p* = 0.00015, Fig. 3A). Similarly, the absolute levels of monounsaturated lipids decreased during Enza treatment (*p* = 0.044; Fig. 3B). Analysis of the different lipid categories revealed a relative decrease in PL, coupled by a relative enrichment of neutral lipids and SL (Fig. 3C).

**Fig. 4:**
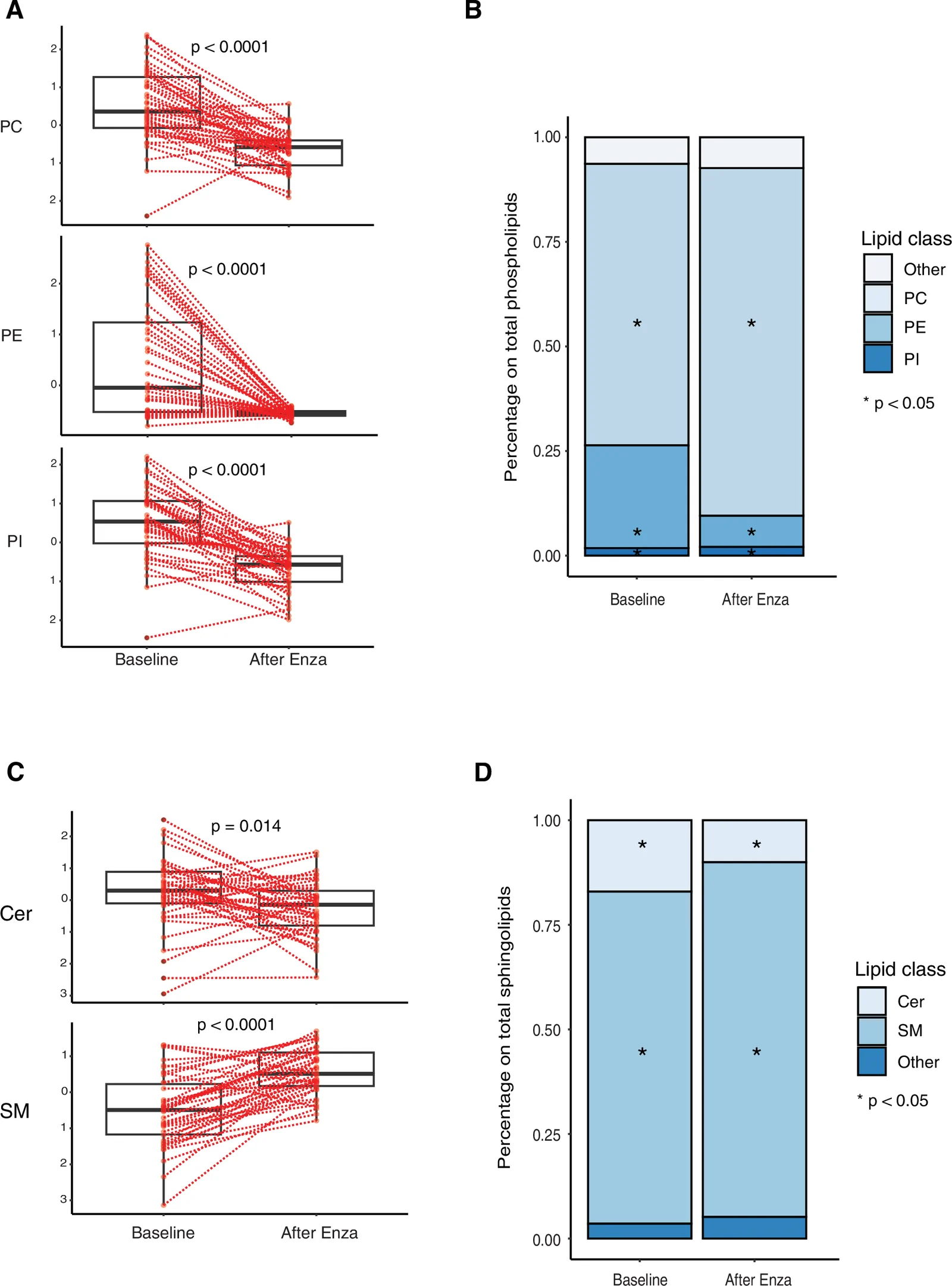
Changes in phospholipid and sphingolipid classes in response to Enza treatment. **A** Paired comparisons showing the absolute levels of phosphatidylcholine (PC), phosphatidylethanolamine (PE), and phosphatidylinositol (PI) before and after enza treatment. **B** Stacked bar plot showing the relative distribution of phospholipid subclasses as a percentage of total phospholipids. **C** Paired comparisons of ceramide (Cer) and sphingomyelin (SM) levels pre- and post-treatment. **D** Stacked bar plot showing the relative composition of sphingolipid subclasses as a percentage of total sphingolipids. Asterisks indicate statistically significant differences (*p* < 0.05).

A significant decrease was observed for all main PL classes (Fig. 4A), with a relative increase of PC over PE species (Fig. 4B). Treatment with Enza was associated with a significant decrease of ceramides and a significant increase of sphingomyelins (SM) (Fig. 4C, D).

### Associations between circulating lipids and survival outcomes in mCRPC patients

The total monounsaturated ceramides assessed after Enza start were significantly associated with PFS in univariate analysis (Hazard ratio [HR] = 1.81, 95% confidence interval [CI] = 1.10–2.70, *p*-value = 0.019) and multivariate analyses (HR = 1.67, 95% CI = 1.01–2.76, *p*-value = 0.044), after adjusting for age, PSA response, Gleason score of the primary tumor, and volume of disease (Supplementary Fig. 3). Notably, monounsaturated ceramides assessed after Enza start significantly correlated with PSA response (Spearman rho = 0.34, *p*-value = 0.027).

A significant association between total SM assessed after Enza start and both PFS and OS was also observed in univariate analysis (PFS HR = 0.60, 95% CI = 0.37 - 0.98, *p* = 0.041: OS HR = 0.53, 95% CI = 0.30–0.94, *p* = 0.029). After multivariate adjustment, total SM concentration remained significantly associated with OS (HR = 0.49, 95% CI = 0.24–0.97, *p* = 0.04), while showing a strong trend towards statistical significance for PFS (HR = 0.49, 95% CI = 0.31–1.01, *p* = 0.053; Supplementary Fig. 3). No significant associations between overall PL or sphingolipid classes at baseline and PFS or OS were found.

**Fig. 5:**
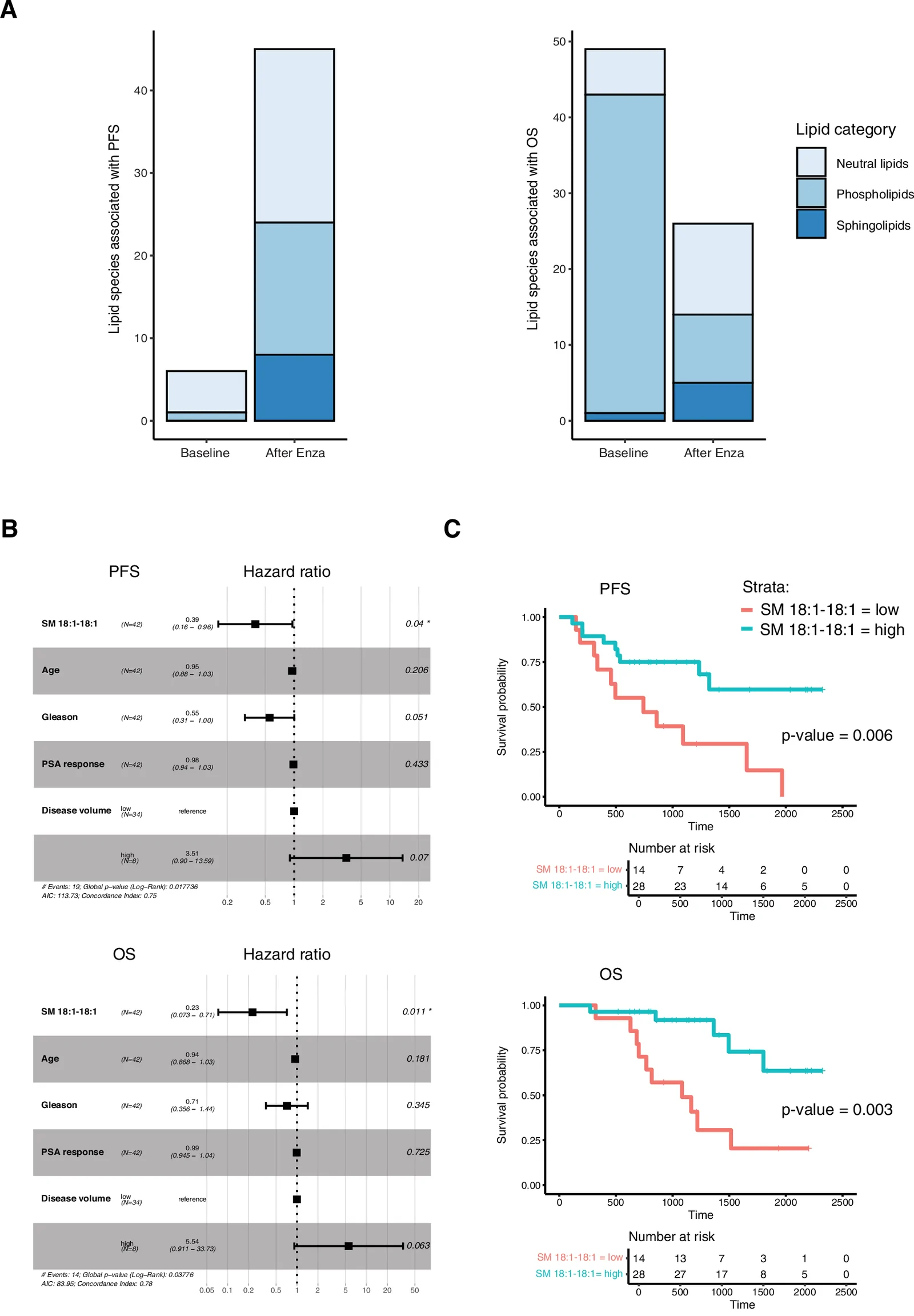
Associations between circulating lipids and survival outcomes in mCRPC patients. **A** Barplot depicting the number of lipid species associated with PFS (left panel) and OS (right panel) at both univariable and multivariable analysis at baseline and after Enza start **B** Forest plots of multivariate Cox regression analyses of SM 18:1-18:1 for progression-free survival (PFS, top) and overall survival (OS, bottom). Higher SM 18:1-18:1 levels were independently associated with improved PFS and OS after adjusting for age, Gleason score, PSA response, and disease volume. **C** Kaplan–Meier survival curves stratified by SM 18:1-18:1 levels (high vs. low) for PFS (top) and OS (bottom). Patients with higher SM 18:1-18:1 levels exhibited significantly better survival outcomes. Patients were categorized as with high levels of SM 18:1–18:1 based on the upper tertile cutpoint. The number at risk is reported below each plot. Time is reported in days from progression of ADT.

Concerning the individual lipid species significant associations between PFS and 6 individual lipid species were observed at baseline and 45 lipid species after Enza treatment in both univariate and multivariate analyses (Fig. 5A; Supplementary Table 3). For OS, significant associations were observed for 49 species assessed at baseline and 26 species assessed after Enza start in both univariate and multivariate analyses (Fig. 5A; Supplementary Table 4). Most associations involved PLs and neutral lipids, reflecting their higher representation among tested species. Notably, SL associations with survival increased from baseline (*n* = 1) to post-treatment (*n* = 13), suggesting a potential link to response to AR-targeted therapy. However, given the limited sample size, along with the high number of analytes, no individual lipid species passed correction for multiple testing through false discovery rate assessment. We then focused on lipid species previously reported to be linked to Enza response in patient-derived explants Ki-67 changes [12]. Of these, the concentration of SM 18:1-18:1 assessed after Enza start was significantly associated with both PFS and OS in univariate (HR PFS = 0.55, 95% CI = 0.31–0.99, *p* = 0.049; HR OS = 0.38, 95% CI = 0.18–0.84, *p* = 0.017) and multivariate analyses (HR PFS = 0.39, 95% CI = 0.16–0.96, *p* = 0.04; HR OS = 0.23, 95% CI = 0.07–0.71, *p* = 0.011; Fig. 5B). Kaplan–Meier curves stratifying patients upon SM 18:1-18:1 concentration assessed after Enza start further confirmed that patients with higher SM 18:1-18:1 level on-treatment had significantly better outcomes compared to patients with lower levels (Fig. 5C). No other significant association between the lipid species reported by Butler et al and PFS or OS in our cohort was observed [12].

## Discussion

Lipid metabolic reprogramming, driven in part by AR-mediated transcriptional control of biosynthetic enzymes, is a recognized hallmark of PCa [19]. While lipidomic changes are well-characterized in primary tumors, the systemic lipidome in advanced stages, particularly mCRPC, remains poorly defined. Limited data on therapy-induced lipidomic shifts hinder understanding of AR-driven metabolic adaptations. Recent studies in both metastatic and localized PCa highlight the central role of lipid metabolism across disease stages [20, 21]. In this context, our study provides a comprehensive characterization of the systemic lipid landscape in patients with mCRPC, integrating longitudinal plasma lipidomic profiling during treatment with clinical outcomes, thereby reinforcing and extending this emerging line of evidence.

Profound alterations in the circulating lipidome of patients with mCRPC were observed, reflecting both tumor and therapy-driven remodeling. Compared to C-FS, mCRPC patients exhibit a distinct enrichment in monounsaturated and long-chain PL.

These changes, consistent with previous findings in primary PCa, suggest increased activity of AR-regulated enzymes such as fatty acid synthase (FASN), stearoyl-CoA desaturase (SCD), and elongases of very long-chain fatty acids (ELOVL) involved in *denovo* lipogenesis, membrane biogenesis, proliferation, and resistance to oxidative stress [22–25]. Moreover, circulating monounsaturated lipid levels also positively correlated with PSA concentrations, reinforcing their association with both tumor burden and clinical disease activity.

Lipid class analysis reveals a shift from energy storage toward membrane biosynthesis, with reduced levels of neutral lipids (e.g., triglycerides, cholesterol esters) and increased phospholipids. The relative enrichment of PE compared to PC within the phospholipid pool suggests a remodeling of membrane composition that prevents ER stress and the unfolded protein response in mCRPC, as well as enhance membrane fluidity. These changes, in part reversed by Enza treatment, could contribute to key malignant features such as increased proliferative capacity, invasive potential, and metastatic ability, ultimately supporting tumor cell adaptation and resistance to therapy. Elevated ceramides, increased in mCRPC, may modulate cellular response to treatment through their impact on apoptosis, cell-survival, and DNA repair regulation [26].

A major strength of our study lies in the longitudinal assessment of the systematic lipidomics during Enza treatment, combined with the clinical homogeneity of the cohort in terms of baseline characteristics and prior treatments, which enhances the reliability and interpretability of the findings.

Enza treatment was followed by a significant reduction of total lipids and monounsaturated fats, indicating suppressed lipid biosynthesis. It also induced coordinated remodeling across lipid classes, with decreased ceramides and major PLs, and increased SM—possibly reflecting a compensatory response to limit ceramide-induced cytotoxicity under AR inhibition [27]. In this context, the altered balance between SM and ceramides observed in our cohort may act as a sensor of cellular stress responses [27]. Moreover, androgen-dependent regulation of sphingolipid signaling has been previously demonstrated in prostate cancer models, supporting a functional interplay between androgen signaling and sphingolipid homeostasis [28, 29]

At radiographic progression, monounsaturated lipids began reverting toward pre- treatment levels, suggesting reactivation of resistance-associated metabolic programs

and highlighting the tumor’s metabolic plasticity. However, the limited sample size warrants further investigation.

The association between SL and clinical outcomes has been documented in previous studies, and these results corroborate these observations [26, 30–34]. Among the sphingolipid species, SM 18:1;18:1 emerged as a lipid with prognostic relevance. This sphingomyelin species is predominantly derived from the extracellular sphingomyelin pool via caveolin-1–mediated trafficking, linking its circulating levels to membrane dynamics and lipid exchange processes [35]. In line with these biological features, and consistent with the findings reported by Butler et al., SM 18:1;18:1 was associated with response to enzalutamide in patient-derived prostate cancer explants [12]. We independently validated that elevated circulating SM 18:1;18:1 during Enza treatment correlates with improved PFS and OS, even after adjusting for clinical covariates. This relationship may reflect a treatment-induced rebalancing of the ceramide–sphingomyelin axis, a pathway known to modulate cell fate decisions in cancer. Higher levels of SM 18:1;18:1 could mitigate the accumulation of bioactive ceramides that drive DNA damage, as we previously reported [15]. Furthermore higher SM 18:1;18:1 level may help stabilize membrane microdomains, supporting a sphingolipid environment that is less permissive to pro-survival and stress-response signaling.

While these results highlight distinct lipid alterations, these may arise both from the host, or the tumor itself. The clear response to Enza, however, strongly suggest a tumor origin reflected in serum lipids. This has been clearly demonstrated in mouse models of PCa [13, 36]. Thus, these findings support plasma lipidomics as an indirect surrogate of tumor metabolic state. Over and above their biomarker value for monitoring disease progression, the circulating lipidome may also inform therapeutic strategies, particularly in patients under AR-targeted therapy. In addition, ongoing trials targeting lipid metabolism further underscore the therapeutic relevance of this pathway in PCa (NCT05743621) [16].

Although many patients in the mCRPC cohort had progressed on ADT monotherapy—no longer recommended as first-line treatment for mCRPC—the lipidomic changes observed following androgen deprivation likely reflect fundamental androgen-regulated metabolic alterations that are maintained under contemporary, intensified therapeutic regimens. Accordingly, the lipid signatures identified in this study may capture intrinsic aspects of advanced PCa biology rather than effects strictly dependent on a specific treatment sequence.

While the study provides novel insights on the dysregulation of lipid metabolism and its potential utility in the clinical management of advanced PCa, some considerations remain. The control cohort was not stratified by clinical parameters, introducing potential variability. The small metastatic cohort size, especially the progressive subgroup, may limit generalizability despite cohort homogeneity. Additionally, unrecorded dietary habits and medications could have influenced lipidomic profiles.

## Conclusion

This study identifies the plasma lipidome as a dynamic and disease stage-specific metabolic signature in mCRPC, both at baseline and during Enza treatment. The observed lipidomic alterations likely reflect tumor biology, AR signaling activity, and adaptive responses to therapy. Among the species identified, SM 18:1–18:1 emerged as a promising prognostic biomarker, with independent associations with clinical outcomes, in line with reports from prior preclinical studies. These findings support the clinical utility of plasma lipidomics as a non-invasive biomarker platform and highlight its potential to inform precision medicine strategies in advanced PCa.

## Supplementary information

Supplementary Figures and Legends

Supplementary_table_1

Supplementary_table_2

Supplementery_table_3

Supplementery_table_4

## Data Availability

Data generated and analyzed during this study are included in this published article and its Supplementary Table 2.

## References

[1] Siegel RL, Kratzer TB, Giaquinto AN, Sung H, Jemal A. Cancer statistics, 2025. CA Cancer J Clin. 2025;75:10–45. 10.3322/caac.21871 PMC11745215 39817679

[2] Tagawa ST, Ramaswamy K, Huang A, Mardekian J, Schultz NM, Wang L, et al. Survival outcomes in patients with chemotherapy-naive metastatic castration-resistant prostate cancer treated with enzalutamide or abiraterone acetate. Prostate Cancer Prostatic Dis. 2021;24:1032–40. 10.1038/s41391-021-00318-3 PMC8616757 33612825

[3] Garje R, Riaz IB, Naqvi SAA, Rumble RB, Taplin ME, Kungel TM, et al. Systemic Therapy in Patients With Metastatic Castration-Resistant Prostate Cancer: ASCO Guideline Update. J Clin Oncol. 2025;43:2311–34. 10.1200/JCO-25-00007 40315400

[4] Tilki D, van den Bergh RCN, Briers E, Van den Broeck T, Brunckhorst O, Darraugh J, et al. EAU-EANM-ESTRO-ESUR-ISUP-SIOG Guidelines on Prostate Cancer. Part II-2024 Update: Treatment of Relapsing and Metastatic Prostate Cancer. Eur Urol. 2024;86:164–82. 10.1016/j.eururo.2024.04.010 38688773

[5] Scher HI, Fizazi K, Saad F, Taplin ME, Sternberg CN, Miller K, et al. Increased survival with enzalutamide in prostate cancer after chemotherapy. N Engl J Med. 2012;367:1187–97. 10.1056/NEJMoa1207506 22894553

[6] Beer, Armstrong TM, Rathkopf DE AJ, Loriot Y, Sternberg CN, Higano CS, et al. Enzalutamide in metastatic prostate cancer before chemotherapy. N Engl J Med. 2014;371:424–33. 10.1056/NEJMoa1405095 PMC4418931 24881730

[7] Barfeld SJ, Itkonen HM, Urbanucci A, Mills IG. Androgen-regulated metabolism and biosynthesis in prostate cancer. Endocr Relat Cancer. 2014;21:57–66. 10.1530/ERC-13-0515 24497572

[8] Zadra G, Photopoulos C, Loda M. The fat side of prostate cancer. Biochim Biophys Acta. 2013;1831:1518–32. 10.1016/j.bbalip.2013.03.010 PMC3766375 23562839

[9] Butler LM, Centenera MM, Swinnen JV. Androgen control of lipid metabolism in prostate cancer: novel insights and future applications. Endocr Relat Cancer. 2016;23:219–27. 10.1530/ERC-15-0556 27130044

[10] Giunchi F, Fiorentino M, Loda M. The Metabolic Landscape of Prostate Cancer. Eur Urol Oncol. 2019;2:28–36. 10.1016/j.euo.2018.06.010 30929843

[11] Flanagan S, Lis RT, Huang Y, Jehane L, Vaselkiv JB, Allott EH, et al. Tumor Cholesterol Synthesis, Statin Use, and Lethal Prostate Cancer. Mol Cancer Res. 2025;23:1025–33. 10.1158/1541-7786.MCR-24-0864 PMC12434729 40762635

[12] Butler LM, Mah CY, Machiels J, Vincent AD, Irani S, Mutuku SM, et al. Lipidomic Profiling of Clinical Prostate Cancer Reveals Targetable Alterations in Membrane Lipid Composition. Cancer Res. 2021;81:4981–93. 10.1158/0008-5472.CAN-20-3863 34362796

[13] Zadra G, Ribeiro CF, Chetta P, Ho Y, Cacciatore S, Gao X, et al. Inhibition of de novo lipogenesis targets androgen receptor signaling in castration-resistant prostate cancer. Proc Natl Acad Sci USA. 2019;116:631–40. 10.1073/pnas.1808834116 PMC6329966 30578319

[14] Falchook G, Infante J, Arkenau HT, Patel MR, Dean E, Borazanci E, et al. First-in-human study of the safety, pharmacokinetics, and pharmacodynamics of first-in-class fatty acid synthase inhibitor TVB-2640 alone and with a taxane in advanced tumors. EClinicalMedicine. 2021;34:100797. 10.1016/j.eclinm.2021.100797 PMC8040281 33870151

[15] Ribeiro CF, Rodrigues S, Bastos DC, Fanelli GN, Pakula H, Foiani M, et al. Blocking lipid synthesis induces DNA damage in prostate cancer and increases cell death caused by PARP inhibition. Sci Signal. 2024;17:eadh1922. 10.1126/scisignal.adh1922 PMC11161871 38593154

[16] Nuzzo PV, Rodrigues S, Ribeiro CF, Teixeira IF, Fanelli GN, Bleve S, et al. Targeting cancer metabolism: Therapeutic potential of the fatty acid synthase (FASN) inhibitors. Crit Rev Oncol Hematol. 2025;214:104910.10.1016/j.critrevonc.2025.10491040846043

[17] Talebi A, de Laat V, Spotbeen X, Dehairs J, Rambow F, Rogiers A, et al. Pharmacological induction of membrane lipid poly-unsaturation sensitizes melanoma to ROS inducers and overcomes acquired resistance to targeted therapy. J Exp Clin Cancer Res. 2023;42:92. 10.1186/s13046-023-02664-7 PMC10114329 37072838

[18] Sweeney CJ, Chen YH, Carducci M, Liu G, Jarrard DF, Eisenberger M, et al. Chemohormonal Therapy in Metastatic Hormone-Sensitive Prostate Cancer. N Engl J Med. 2015;373:737–46. 10.1056/NEJMoa1503747 PMC4562797 26244877

[19] Mah CY, Nassar ZD, Swinnen JV, Butler LM. Lipogenic effects of androgen signaling in normal and malignant prostate. Asian J Urol. 2020;7:258–70. 10.1016/j.ajur.2019.12.003 PMC7385522 32742926

[20] Lin HM, Scheinberg T, Portman N, Kim RMN, Mellor R, Huynh K. al. Association of the circulating lipid panel, PCPro, with clinical outcomes in metastatic hormone-sensitive prostate cancer: post hoc analysis of the ENZAMET phase III randomised trial (ANZUP 1304). Ann Oncol. 2025;36:1068–77. 10.1016/j.annonc.2025.05.529 40403846

[21] Lin HM, Yang X, Centenera MM, Huynh K, Giles C, Dehairs J, et al. Circulating Lipid Profiles Associated With Resistance to Androgen Deprivation Therapy in Localized Prostate Cancer. JCO Precision Oncology. 2024;8:2400260. 10.1200/PO.24.00260 39074346

[22] Centenera MM, Scott JS, Machiels J, Nassar ZD, Miller DC, Zinonos I, et al. ELOVL5 Is a Critical and Targetable Fatty Acid Elongase in Prostate Cancer. Cancer Res. 2021;81:1704–18. 10.1158/0008-5472.CAN-20-2511 33547161

[23] Swinnen JV, Brusselmans K, Verhoeven G. Increased lipogenesis in cancer cells: new players, novel targets. Curr Opin Clin Nutr Metab Care. 2006;9:358–65. 10.1097/01.mco.0000232894.28674.30 16778563

[24] Zadra G, Loda M. Metabolic Vulnerabilities of Prostate Cancer: Diagnostic and Therapeutic Opportunities. Cold Spring Harb Perspect Med. 2018;8:a030569. 10.1101/cshperspect.a030569 PMC6169980 29229664

[25] Swinnen JV, Roskams T, Joniau S, Van Poppel H, Oyen R, Baert L, et al. Overexpression of fatty acid synthase is an early and common event in the development of prostate cancer. Int J Cancer. 2002;98:19–22. 10.1002/ijc.10127 11857379

[26] Lin HM, Huynh K, Kohli M, Tan W, Azad AA, Yeung N, et al. Aberrations in circulating ceramide levels are associated with poor clinical outcomes across localised and metastatic prostate cancer. Prostate Cancer Prostatic Dis. 2021;24:860–70. 10.1038/s41391-021-00338-z PMC8387438 33746214

[27] Ogretmen B. Sphingolipid metabolism in cancer signalling and therapy. Nat Rev Cancer. 2018;18:33–50. 10.1038/nrc.2017.96 PMC5818153 29147025

[28] Dayon A, Brizuela L, Martin C, Mazerolles C, Pirot N, Doumerc N, et al. Sphingosine kinase-1 is central to androgen-regulated prostate cancer growth and survival. PLoS One. 2009;4:8048. 10.1371/journal.pone.0008048 PMC2779655 19956567

[29] Portman N, Mak B, Yeung N, Lin HM, Kim RMN, Mellor R, et al. Characterising the effect of circulating sphingolipids on metastatic prostate cancer cells. EBioMedicine. 2025;123:106058. 10.1016/j.ebiom.2025.106058 PMC12741386 41365058

[30] Lin HM, Mahon KL, Weir JM, Mundra PA, Spielman C, Briscoe K, et al. A distinct plasma lipid signature associated with poor prognosis in castration-resistant prostate cancer. Int J Cancer. 2017;141:2112–20. 10.1002/ijc.30903 28741687

[31] Mak B, Lin HM, Kwan EM, Fettke H, Tran B, Davis ID, et al. Combined impact of lipidomic and genetic aberrations on clinical outcomes in metastatic castration-resistant prostate cancer. BMC Med. 2022;20:112. 10.1186/s12916-022-02298-0 PMC8953070 35331214

[32] Cattrini C, Manfredi M, Barboro P, Ghirimoldi M, Mennitto A, Martini V, et al. Untargeted lipidomics reveal association of elevated plasma C18 ceramide levels with reduced survival in metastatic castration-resistant prostate cancer patients. Sci Rep. 2023;13:17791. 10.1038/s41598-023-44157-9 PMC10585001 37853018

[33] Wang Y, Jacobs EJ, Carter BD, Gapstur SM, Stevens VL. Plasma Metabolomic Profiles and Risk of Advanced and Fatal Prostate Cancer. European Urology Oncology. 2021;4:56–65. 10.1016/j.euo.2019.07.005 31378665

[34] Scheinberg T, Lin HM, Fitzpatrick M, Azad AA, Bonnitcha P, Davies A, et al. PCPro: a clinically accessible, circulating lipid biomarker signature for poor-prognosis metastatic prostate cancer. Prostate Cancer Prostatic Dis. 2024;27:136–43. 10.1038/s41391-023-00666-2 PMC10876475 37147359

[35] Vykoukal J, Fahrmann JF, Gregg JR, Tang Z, Basourakos S, Irajizad E, et al. Caveolin-1-mediated sphingolipid oncometabolism underlies a metabolic vulnerability of prostate cancer. Nat Commun. 2020;11:4279. 10.1038/s41467-020-17645-z PMC7453025 32855410

[36] Zadra G, Loda M. When fat goes down, prostate cancer is on the ropes. Mol Cell Oncol. 2019;6:1595308. 10.1080/23723556.2019.1595308 PMC6512936 31131311

