# Supplementary material for: Lipidomic profiling in metastatic prostate cancer captures tumor metabolic rewiring and its modulation by androgen receptor–targeting therapy
Source: Prostate Cancer Prostatic Dis. 2026 Mar 13;29(4):826–35. doi: 10.1038/s41391-026-01100-z (PMC13139911; doi:10.1038/s41391-026-01100-z)
Supplement: Supplementary file 1 [file 41391_2026_1100_MOESM1_ESM.pdf]

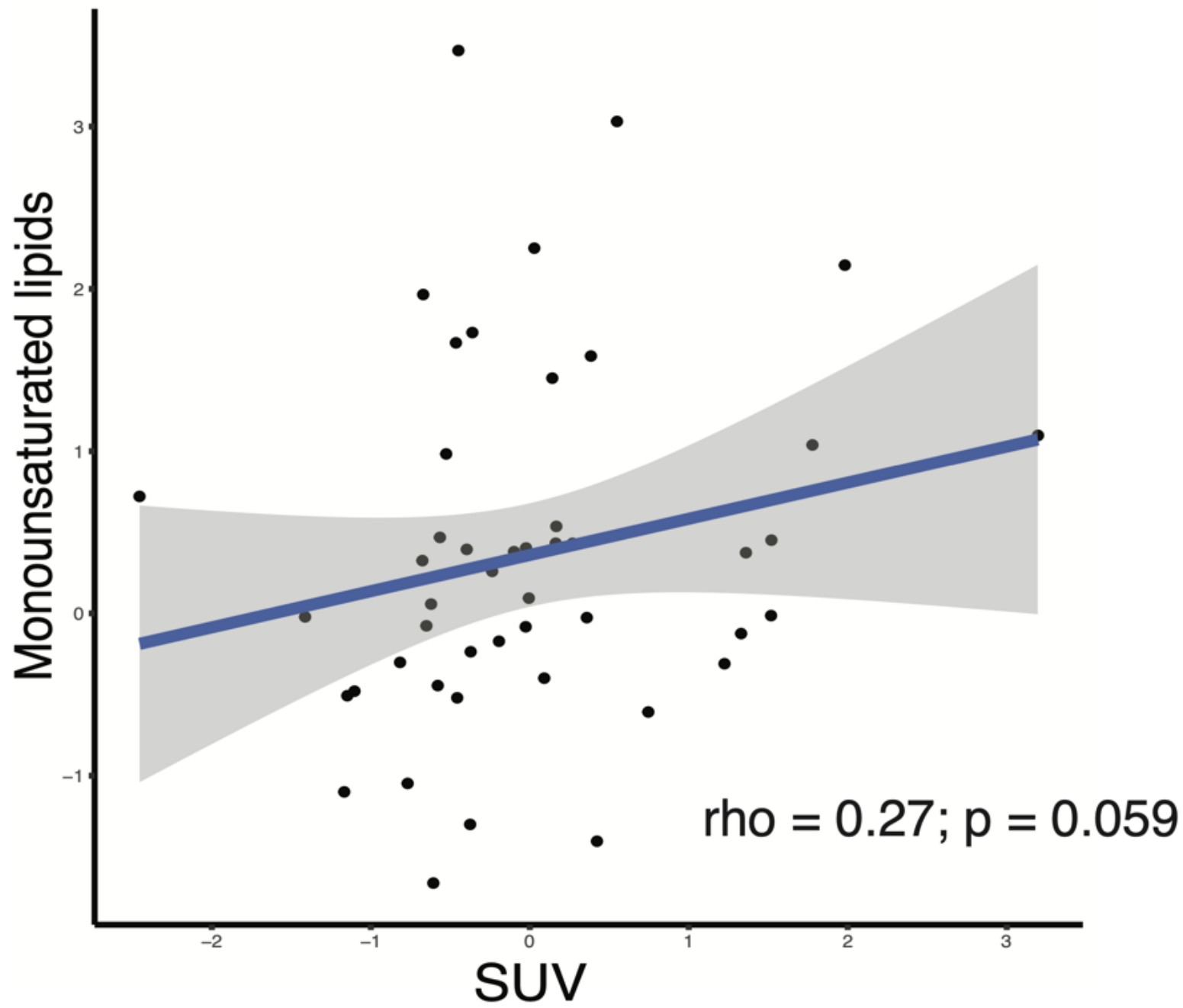

Supplementary Figure 1. Correlation of Monounsaturated lipid levels with SUV assessed before the start of Enza in mCRPC patients with PET-PSMA. Monounsaturated lipid and SUV are reported after log-transformation and scaling.

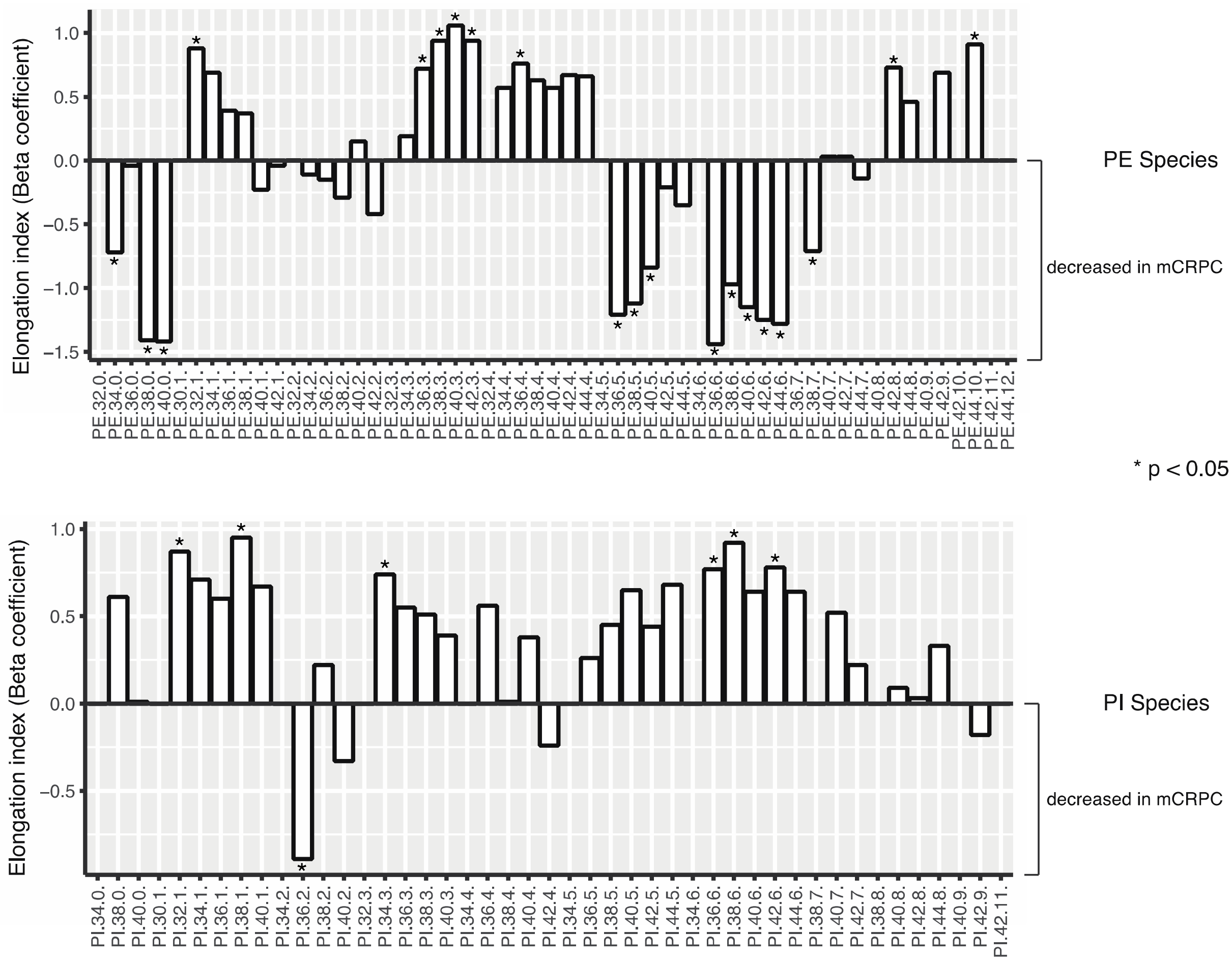

Supplementary Figure 2: Lipid elongation of PE (upper panel) and PI (lower panel) species in mCRPC compared to C-FC. Lipid elongation of each PE and PI species was expressed in terms of elongation indexes. Briefly, the abundance of each species was divided by the shortest species of the same saturation subclass. Linear models were used to compare elongation indexes between mCRPC and C-FS, adjusting for Age and BMI. On the y-axis are reported the beta coefficients of the linear models used to perform the differential analysis.

Monounsaturated Cer and PFS

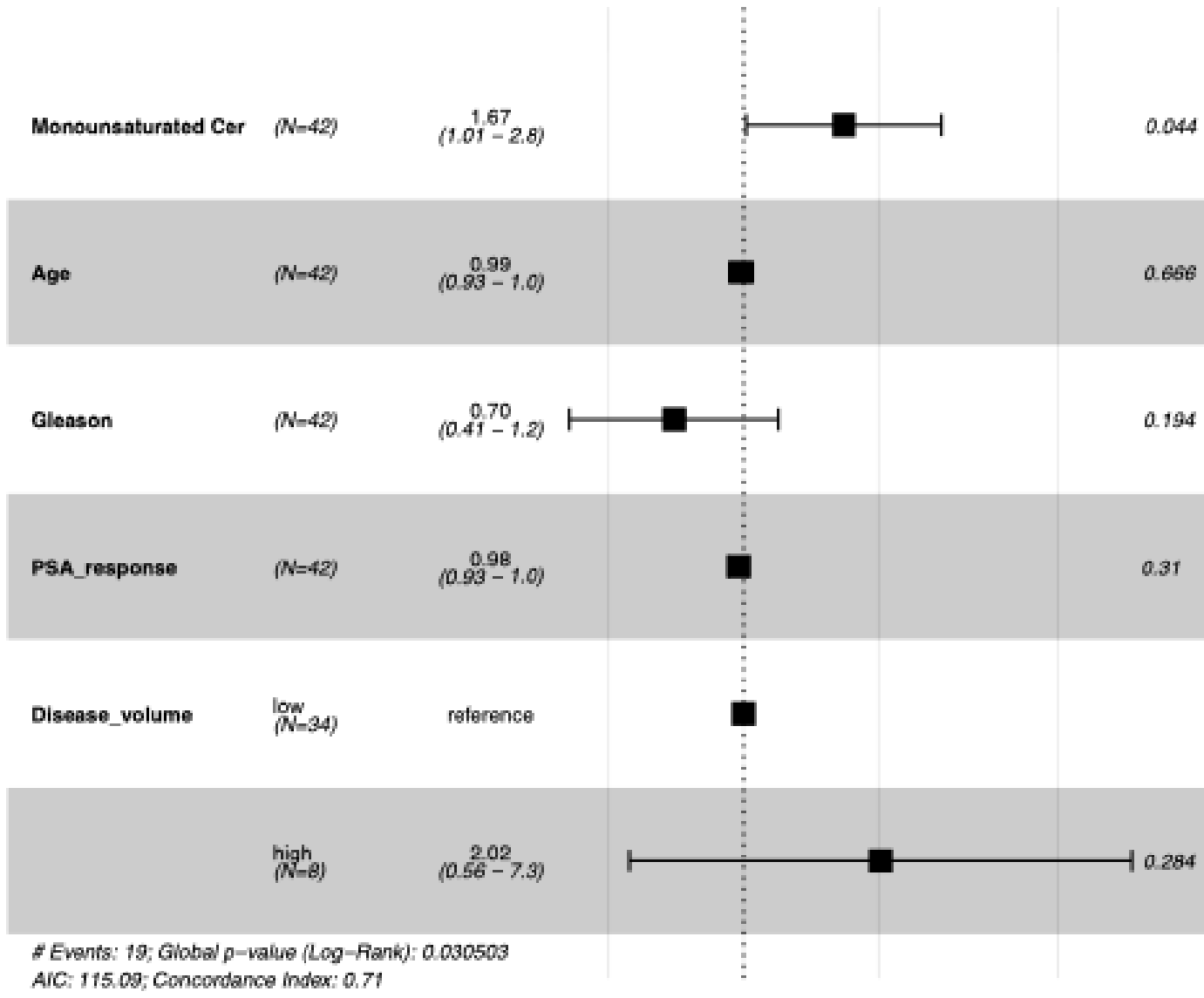

Total SM and PFS

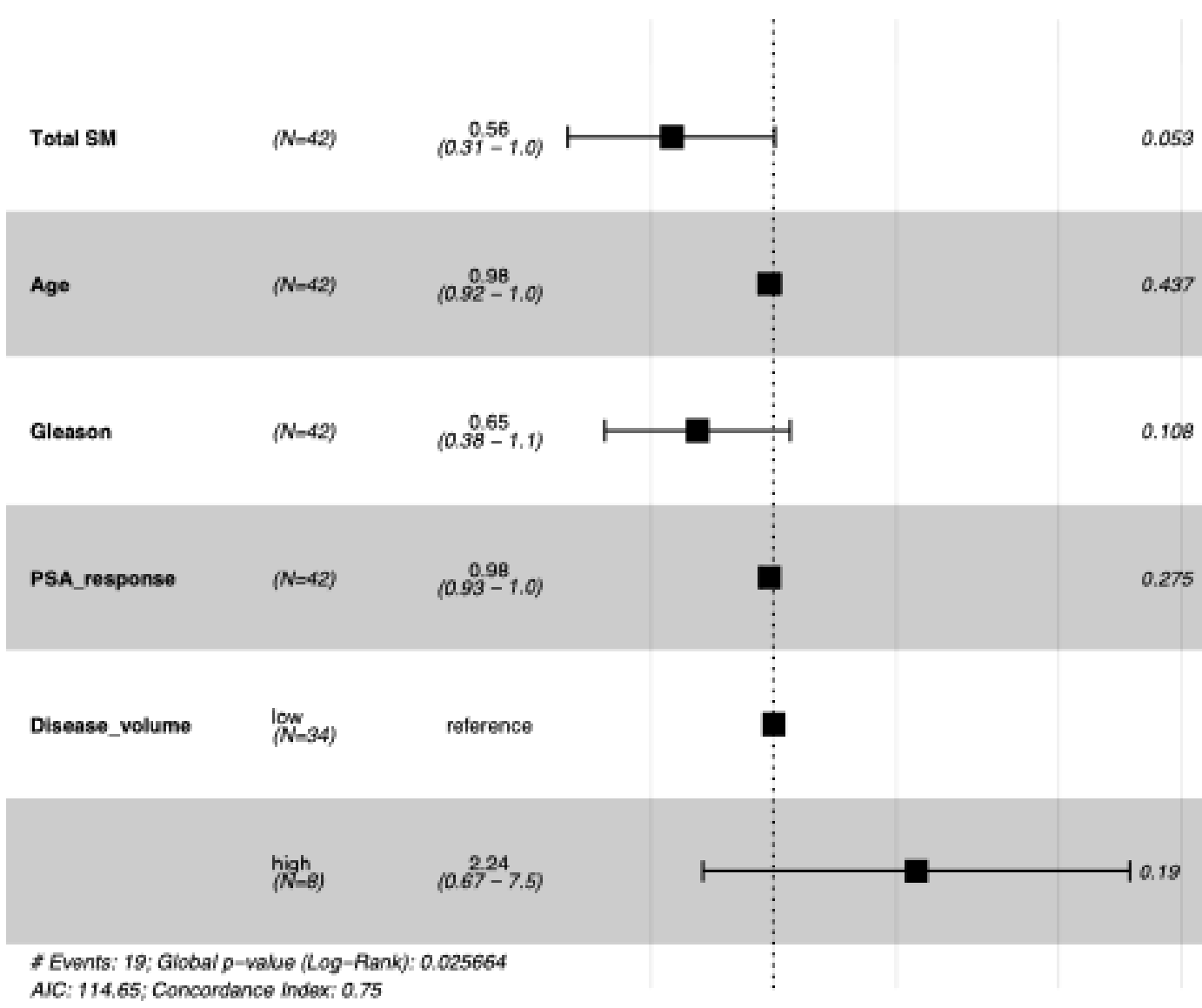

Total SM and OS

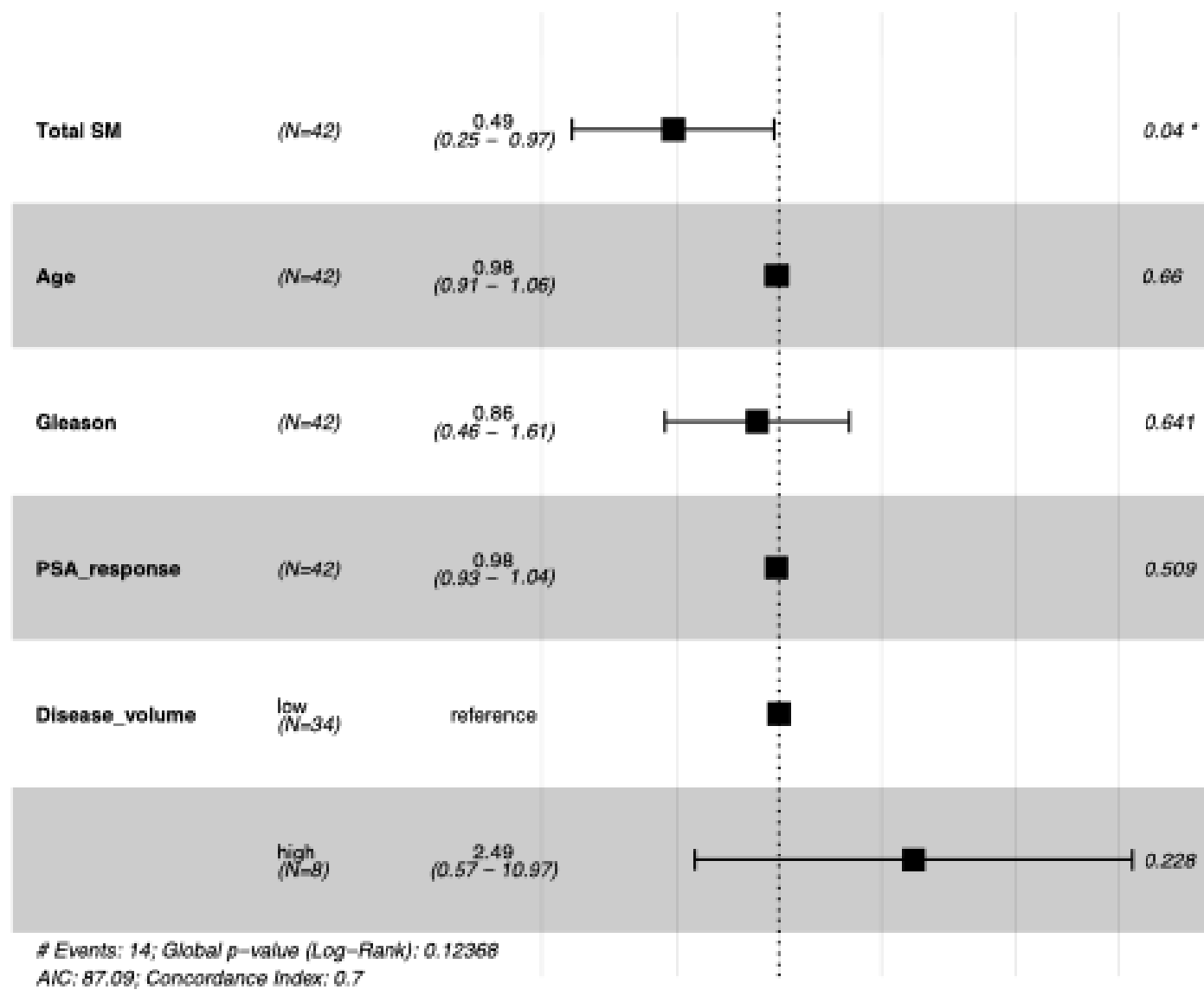

Supplementary Figure 3: Multivariable survival analysis of monounsaturated ceramides and total sphingomyelins assessed in mCRPC patients after the start of Enza. The upper panel refers to Monounsaturated Ceramides and progression-free survival, the medium one to total sphingomyelins and progression, and the lower one to total sphingomyelins and overall survival.

Supplementary tables legends

Supplementary table 1. Complete patients' metadata.

Supplementary table 2. Raw lipidomics data. Spreadsheet 1 includes sample legend. Spreadsheet 2 includes lipidomics data on the individual lipid species. Spreadsheet 3 includes lipidomics data in the fatty acid notation as provided by Lipometrix. The number of carbons and unsaturations of the attached fatty acid are reported for each item.

Supplementary table 3. Lipid species significantly associated with PFS in univariable and multivariable analyses.

Supplementary table 4. Lipid species significantly associated with OS in univariable and multivariable analyses.
